# Improvement of peptide identification with considering the abundance of mRNA and peptide

**DOI:** 10.1186/s12859-017-1491-5

**Published:** 2017-02-16

**Authors:** Chunwei Ma, Shaohang Xu, Geng Liu, Xin Liu, Xun Xu, Bo Wen, Siqi Liu

**Affiliations:** BGI-Shenzhen, Shenzhen, 518083 China

**Keywords:** Bioinformatics, Mass spectrometry, RNA-Seq, Machine learning, Shotgun proteomics, Proteogenomics

## Abstract

**Background:**

Tandem mass spectrometry (MS/MS) followed by database search is a main approach to identify peptides/proteins in proteomic studies. A lot of effort has been devoted to improve the identification accuracy and sensitivity for peptides/proteins, such as developing advanced algorithms and expanding protein databases.

**Results:**

Herein, we described a new strategy for enhancing the sensitivity of protein/peptide identification through combination of mRNA and peptide abundance in Percolator. In our strategy, a new workflow for peptide identification is established on the basis of the abundance of transcripts and potential novel transcripts derived from RNA-Seq and abundance of peptides towards the same life species. We demonstrate the utility of this strategy by two MS/MS datasets and the results indicate that about 5% ~ 8% improvement of peptide identification can be achieved with 1% FDR in peptide level by integrating the peptide abundance, the transcript abundance and potential novel transcripts from RNA-Seq data. Meanwhile, 181 and 154 novel peptides were identified in the two datasets, respectively.

**Conclusions:**

We have demonstrated that this strategy could enable improvement of peptide/protein identification and discovery of novel peptides, as compared with the traditional search methods.

**Electronic supplementary material:**

The online version of this article (doi:10.1186/s12859-017-1491-5) contains supplementary material, which is available to authorized users.

## Background

Mass spectrometry (MS)-based methods have become a powerful and main means for identifying peptides/proteins in proteomics studies. Generally, the acquired MS/MS data from mass spectrometry is analyzed with the software and searched against protein sequence databases for protein identification. Several such software are available, commercial or freely available, such as SEQUEST [1], MASCOT [2], X!Tandem [3], OMSSA [4], MyriMatch [5] and MS-GF+ [6]. Generally, the algorithms development in these software aim at improving the estimation scores that evaluate the extent of peptide spectrum match (PSM) and reflect the quality of the cross-correlation between the experimental and the theoretical data. In general, the better the two datasets are matched, the higher scores are achieved. The top rank PSM is not necessarily correct, however, due to flaws of scoring algorithm or poor quality of MS/MS spectrum. Hence, correct match is introduced using a target-decoy search model to estimate a false discovery rate (FDR). Although sophisticated algorithms for annotation of mass spectra have dramatically developed, the identification rate to peptides/proteins upon MS/MS data is still not so satisfied yet, because a poor identification of peptides/proteins is related with many causal factors, such as low efficiency of peptide ionization, low-quality or noisy MS/MS spectra, dynamic range of protein abundances, the complexity of protein samples and flaws of scoring algorithm.

There are two method categories that are developed to improve the sensitivity of peptide/protein identification upon MS/MS data. One is the post-processing algorithm that is designed to validate and filter PSMs based on the search engine’s results, such as PeptideProphet derived from the empirical modeling [7], Percolator comes from the semi-supervised learning [8] and IPeak based on the multi-search engines [9]. These algorithms usually incorporate additional information from the MS/MS experiments for re-scoring PSMs, such as retention time of peptide chromatography, peptide charge state, or mass accuracy. Another one is the algorithms to utilize external information, i.e. the information gained from the non-MS/MS-based experiments, such as RNA-Seq data [10–12]. Recently Wang et al. described an approach to utilize the mRNA abundance to limit the sizes of protein sequence databases as to improve the sensitivity of protein identification [13]. Meanwhile, Avinash et al. proposed a method to utilize RNA-Seq and GPMDB protein observation frequency to rescore or adjust the protein identification probabilities as to augment the identification sensitivity, even though its application was restricted at protein but not at peptide or PSM level [14]. Also Wu et al. described a novel bioinformatics workflow to focus on the identification of new peptides which were not present in the standard protein databases but in the datasets derived from RNA-Seq data [15]. This workflow, however, doesn’t utilize the abundance information from the RNA-Seq data to assist in peptide identification. Though many efforts have been devoted to the two categories, there is lack of method that enables combination of the advantages from both methods.

In this work, we introduced a novel workflow of proteomic analysis by integration of the post-processing algorithm and the external information gained from RNA-Seq data. Through incorporating the abundance of mRNAs and peptides for rescoring PSMs, and the potential novel transcript sequences, we demonstrated the sensitivity of peptide/protein identification and discovery of novel peptides to be significantly improved in the new type of pipeline.

## Methods

### Datasets

The two MS/MS datasets were used in this study, the MS/MS data for Jurkat cell line and mouse liver tissues generated by LTQ Orbitrap velos. The raw data were downloaded from the PeptideAtlas (http://www.peptideatlas.org/) or iProx (http://www.iprox.cn) data repository with the identifier PASS00215 or IPX00003601 (ftp://211.102.209.248/IPX00003600/IPX00003601/). The paired end 200 bp sequencing RNA-Seq data for the Jurkat cell line generated from Illumina HiSeq 2000 was downloaded at NCBI’s Gene Expression Omnibus (GEO) repository with accession number GSM1104129 [16]. The paired-end 90 bp sequencing RNA-Seq data for mouse liver tissue generated by Wu et al. was downloaded from the Short Read Archive under study accession number SRP033468 [15].

### RNA-Seq data processing

The analysis of RNA-Seq data was conducted under the Trapnell’s protocol [17]. For Jurkat cell line, the sequence reads were mapped to the Ensembl human genome (release GRCh37.75) using Tophat (version 2.0.8). Transcriptome reconstruction and expression quantification were implemented by Cufflinks (version 2.2.1). For mouse liver, the parameters for Tophat and Cufflinks were followed by that suggested by Wu et al. [15]. Fragments Per Kilobase of transcript per Million mapped reads (FPKM) was used for estimation of the transcriptional abundance for each transcript [18]. Basically, the original data gained from pair-end sequencing was input into Cufflinks, and the transcript abundance was estimated with the optimized parameters in the program (The detailed scripts to generate the FKPM values were presented in Additional file 1).

### The customized protein sequence database

After getting mapping result from Tophat, CustomProDB (version 1.7.0) was used to construct a customized protein database. In the customized database, an identified protein with its corresponding FPKM less than 0.1 was filtered out. Novel transcripts were constructed by Cufflinks, and further compared with reference annotation using Cuffcompare (version 2.2.1), in which transcripts labeled with j stand potentially novel isoforms (fragments), and with u represent unknown, intergenic transcripts. The translated peptides with the longest frame were added into the customized database.

### Peptide search upon MS/MS data

The raw MS/MS data were converted into MGF and mzXML format by using msconvert in ProteoWizard software package (v. 3.0.5047). Mascot (version 2.3.02) was employed for peptide search upon MS/MS data against Ensembl human proteome database (release GRCh37.75) and the customized database, respectively. Trypsin was specified as the enzyme with a maximum of two missed cleavages. For the two datasets, precursor mass tolerance was set at 10 ppm, and fragment ion mass tolerance at 0.05 Da for Jurkat and at 0.5 Da for mouse liver. Carbamidomethylation of cysteine was set as a fixed modification, and oxidation of methionine was set as a variable modification. The automatic Mascot decoy database search was performed. The results of Mascot were processed by MascotPercolator (v2.07) [8, 19]. The q-value for identification was set to 1% at PSM or peptide level.

### Peptide identification through integrating the abundance of peptides and transcripts

The abundance for each peptide based on extracted ion chromatogram (XIC) was estimated by a tool developed in-house (more details are described in Additional file 2). The transcript abundance was directly derived from the RNA-Seq data analysis. For a transcript that well matched with protein database, its abundance was directly assigned a feature in rescoring PSM, for a sequence from decoy database, a randomly selected transcript abundance was assigned, and for an un-transcribed sequence, the transcript abundance was assigned as zero. The two sets of quantitative features were taken by Percolator that is an efficacious semi-supervised learning method for rescoring of database searching result [19]. To avoid overfitting, Percolator randomly splits the PSMs into three subsets and trains three separate SVM classifiers, each trained on two of the three subsets and tested on the remaining subset [20]. In addition, in total there are 47 features derived from MascotPercolator output were also used for rescoring PSMs and were shown in (see Additional file 3: Figure S1). The detailed parameters and command line used for Percolator are presented in Additional file 1.

## Results and Discussion

### Construction of the customized database

In a cell, a part of the genome encoding genes is transcriptional. It is reasoned that the transcripts at very low level could be translated to limited proteins, which are unlikely to be easily detected. With RNA-Seq technology, the transcript abundance in cells and tissues could be measured more accurately and reproducible, while FPKM value is assumed as a criterion to evaluate the unexpressed or lowly expressed genes. The detailed scripts to generate the FKPM values were presented in Additional file 1. The total number of reads and alignment rate for the datasets used in this study were presented in (see Additional file 4: Table S1). Firstly, we constructed the reduced protein sequence databases by eliminating the proteins whose corresponding transcript expressions were below the selected threshold. Based on the RNA-Seq data obtained from Jurkat cell line, 104763 entries in the Ensembl human protein database were shrunk to 71021 entries using CustomProDB, while 8909 potential polypeptides were found from the potential novel transcripts. Thus, the customized database for Jurkat cell line comprised 79930 entries. With the similar strategy, the customized database for mouse liver contained 31843 entries including 6099 potential polypeptides. As shown in Table 1, before filtering low-RNA-level protein entries, the identified peptides in peptide level FDR less than 1% were 71499 and 50083 for Jurkat cell line and mouse liver, while after the filtering, the values became 72283 and 50993 with slight increase of 1.10% and 1.82%, respectively. Specifically, 71043 and 49607 peptides were identified by the both methods in Jurkat cell line and mouse liver datasets, respectively. Impressively, 66 novel peptides were identified in the customized database in Jurkat cell line without filtering low-RNA-level protein entries, whereas 105 novel peptides were found in the customized database after the filtering. In the mouse liver, the similar results were achieved, indicating that 76 novel peptides were identified in the customized database without filtering the low-RNA-level protein entries, whereas 116 new peptides were detected in the reduced database. It was obvious that the reduced and customized database through eliminating the proteins with the corresponding transcript at low level could slightly improve the identification sensitivity to peptides. In summary, up to 2.11% improvement of peptide identification could be achieved with searching the MS/MS data against the customized protein database (Table 1, method: DB_ref_ + DB_novel_ + R_low_) as compared with the reference protein database (Table 1, method: DB_ref_).

**Table 1:** Summary of peptide identification with 1% FDR in peptide level for different methods on two data sets

No.	Methods	Jurkat cell line		Mouse liver	
		Peptide	Improvement	Peptide	Improvement
1	DB_ref_	71645	-	49937	-
2	DB_ref_ + DB_novel_	71499	-	50083	-
3	DB_ref_ + DB_novel_ + R_low_	72283	1.10%	50993	1.82%
4	DB_ref_ + DB_novel_ + R_low_ + F_mRNA_	75649	5.80%	52503	4.83%
5	DB_ref_ + DB_novel_ + R_low_ + F_peptide_	76259	6.66%	52170	4.17%
6	DB_ref_ + DB_novel_ + R_low_ + F_peptide+mRNA_	77682	8.65%	53024	5.87%

Note:

1. DB_ref_ : searching MS/MS data against with the reference protein database and then using MascotPercolator to process the identification results

2. DB_ref_ + DB_novel_ : searching MS/MS data against with the reference protein database adding the novel transcript-derived proteins, and then using MascotPercolator to process the identification results

3. DB_ref_ + DB_novel_ + R_low_ : searching MS/MS data against with the customized protein database (reference proteins + novel transcript-derived proteins + removing low-RNA-level protein entries), and then using MascotPercolator to process the identification results

4. DB_ref_ + DB_novel_ + R_low_ + F_mRNA_ : searching MS/MS data against with the customized protein database (reference proteins + novel transcript-derived proteins + removing low-RNA-level protein entries), and then using MascotPercolator to process the identification results with adding the transcript abundance as a feature (F_mRNA_)

5. DB_ref_ + DB_novel_ + R_low_ + F_peptide_: searching MS/MS data against with the customized protein database (reference proteins + novel transcript-derived proteins + removing low-RNA-level protein entries), and then using MascotPercolator to process the identification results with adding the peptide abundance (MS1 XIC of peptide) as a feature (F_peptide_)

6. DB_ref_ + DB_novel_ + R_low_ + F_peptide+mRNA_: searching MS/MS data against with the customized protein database (reference proteins + novel transcript-derived proteins + removing low-RNA-level protein entries), and then using MascotPercolator to process the identification results with adding the two features (F_peptide+mRNA_ = F_mRNA_ + F_peptide_)

### Improvement of peptide identification on account of transcript abundance

**Fig. 1:**
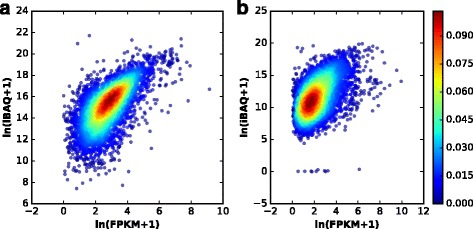
The correlation of transcript and protein abundances. (**a**) Jurkat cell line dataset and (**b**) mouse liver dataset. The Pearson correlation coefficients were 0.6318 and 0.4987 for Jurkat cell line and mouse liver datasets, respectively. Intensity based absolute quantification (iBAQ) was utilized to represent the protein abundance

**Fig. 2:**
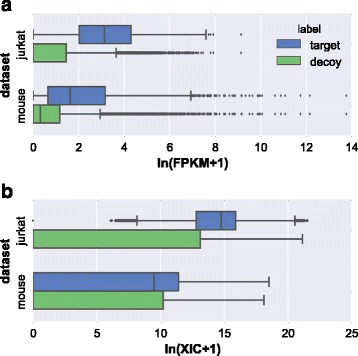
The distribution of two features (XIC and FPKM) in target and decoy PSMs. The value of the two features are log transformed

**Fig. 3:**
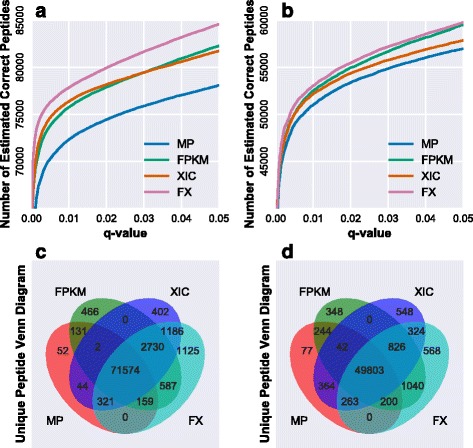
Peptide identification versus different q-values and Venn plot for peptide identification. (**a**) and (**b**) Graphs display the estimated number of correct peptides for the Jurkat cell line and mouse liver data sets. (**c**) and (**d**) Unique peptide Venn plots for four methods. “MP” stands for processing by MascotPercolator, FPKM stands for processing by MascotPercolator adding FPKM as feature, XIC stands for processing by MascotPercolator adding XIC as feature, XIC + FPKM and FX stand for processing by MascotPercolator adding both FPKM and XIC as features

It is generally accepted that the transcript abundance is not well correlated with the protein abundance in cell or tissue. However, many studies have reported that the abundance between RNA and protein levels are positively correlated and it is a reasonable assumption that proteins corresponding to high-abundance transcripts are more likely to be found in a sample [21, 22]. The abundance correlations of mRNA and protein in Jurkat cell line and mouse liver are basically positive with correlation efficiencies at 0.6318 and 0.4987 (Fig. 1a and b), respectively. In this study, we postulated transcript abundance as a feature to impact the peptide or protein identification. Hence, transcript abundance was taken as a feature in Percolator processing. For a protein, its corresponding transcript abundance was obtained from the RNA-Seq data. If a transcript was undetectable, then the feature value was assigned zero, while if a PSM was matched to more than one protein, the largest transcript abundance was taken as the feature value. As regards the two RNA-Seq data sets described above, Jurkat cell line and mouse liver, the features for transcript abundance (FPKM) and peptide abundance (MS1 XIC) for target PSMs and decoy PSMs were shown in Fig. 2, indicating that the feature for either transcript or peptide in target PSMs was distinct from that in decoy PSMs, however, the feature values appeared a large diversity. With inputting the transcript abundance into Percolator processing, the peptide identified were 75649 for Jurkat cell line and 52503 for mouse liver, respectively. As compared with the identification results in Fig. 3, the total peptides identified was increased approximately 5%, in which the overlap rates were about 95% for Jurkat cell line and 96% for mouse liver. Of the mis-overlapped peptides, 3783 peptides for Jurkat cell line and 2214 for mouse liver were only identified through the treatment of transcript abundance, whereas 417 peptides for Jurkat cell line and 704 for mouse liver were merely detected without such treatment. The comparison for the identified peptides strongly suggested that transcript abundance was a useful feature to benefit peptide identification.

### Improvement peptides identification on account of MS1 XIC

MS1 XIC areas for peptide MS1 spectra corresponding to peptide identification events were generally extracted from corresponding RAW data files, and were treated as an indicator for peptide abundance. We considered MS1 XIC as a feature to enhance the peptide identification rate, and took it into Percolator processing. By processing the same datasets with MS1 XIC as a feature, the peptides identified were 76259 for Jurkat cell line and 52170 for mouse liver, respectively. Compared the data without MS1 XIC treatment, the identification rate was improved to about 6.7% for Jurkat cell line and 4.2% for mouse live due to introducing the new feature. In the identified peptides through with/without MS1 XIC treatment, approximately 94% of them for Jurkat cell line and 97% for mouse liver were overlapped. As for the mis-overlapped peptides, 4318 for Jurkat cell line and 1698 for mouse liver were specifically identified after inputting MS1 XIC, while 342 and 521 for the two species were uniquely perceived under without MSI XIC treatment. These results hence endorsed our postulation that the MS1 XIC feature can benefit peptide identification in Percolator processing.

### Further improvement of peptide identification with the combined features

**Fig. 4:**
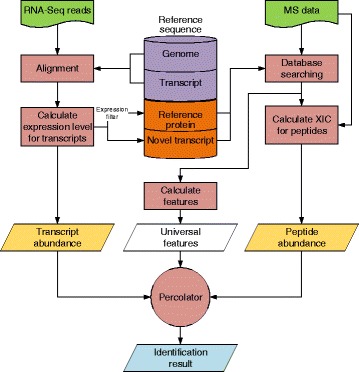
Workflow

As adding each feature, FPKM or MS1 XIC, into Percolator did improve peptide identification rate, a proposal was naturally raised if the combination of the two features could further enhance the rate. We integrated both FPKM and MS1 XIC features into Percolator as the protocol illustrated in Fig. 4, and re-searched the MS/MS data upon the same two datasets described above. Total identified peptides by the treatment were 77682 for Jurkat cell line and 53024 for mouse liver, with 8.7% and 5.9% increase compared without addition of the features, which almost completely covered the peptides identified without the treatment (Fig. 3). Moreover, the specifically identified peptides after the treatment were 5628 for Jurkat cell line and 2758 for mouse liver, which the corresponding peptides derived from the un-treatment were only 229 and 727, respectively. Furthermore, we investigated the quality of uniquely identified 402 peptides by the method with adding MS1 XIC eature but not for the method with adding both FPKM and MS1 XIC features, and a comparison of Mascot scores for peptide identification towards all the peptides identified in the dataset was presented in (Additional file 5: Figure S2). The results demonstrated large portion of the 402 peptides with very low scores, indicating their unsatisfied quality of identification. According to the above results, we concluded that the number of identified peptides can be significantly improved with simultaneously using FPKM and MS1 XIC as the features for post-processing by Percolator. In addition, the 183 novel peptides for Jurkat cell line and 154 for mouse liver came from the RNA-Seq derived database with filtering proteins at low transcript abundance. In previous study, Shanmugam et al. found that the sensitivity of protein identification could improved by using RNA-Seq and GPMDB protein observation frequency. In their study, the probability adjustment of identification was limited at protein level but not for peptide, and the potential novel sequences from RNA-Seq data was not fully utilized. Besides, they combined the two features for probability adjustment, RNA-Seq and GPMDB protein observation frequency, and observed no remarkable improvement after the treatment. In addition, we compared the results of our approach with that of the method (building customized database from RNA-Seq data) similar with previous study [13]. The details of building the customized database were described above. Our approach identified 7.47% more peptides and 2.54% more proteins (77682 peptides, 6415 proteins) than the previous method (72283 peptides, 6256 proteins) on Jurkat cell line dataset. And our approach identified 3.98% more peptides and 0.46% more proteins (53024 peptides, 5010 proteins) than the previous method (50993 peptides, 4987 proteins) on mouse liver dataset. The results in PSM level were similar with that in peptide level as shown in (Additional file 6: Table S2). We also systematically evaluated these features importance based on the weighting scores generated from Percolator. The evaluation results have been presented in (see Additional file 3: Figure S1). The results strongly indicated that the two features offered higher weight than most of other features used in Percolator processing. Our data as shown above demonstrated the identification rates were indeed improved in either individual feature or combined features, suggesting that the two features, FPKM and MS1 XIC, were properly selected for improvement of peptide identification, especially in Percolator.

As for building customized database (step 1) and adding the two features into Percolator processing (step 2), in order to further explorer which step is more important, when only performed the step 1, as shown in Table 1 (Method: DB_ref_ + DB_novel_ + R_low_), 72283 peptides were identified and this is 0.89% more peptide identification than the standard approach (taken reference protein as the database) (71645 peptides) on Jurkat cell line dataset. There were 50993 peptides identified and this is 2.11% more peptide identification than the standard approach (taken reference protein as the database) (49937 peptides) on mouse liver dataset. When performed the step 1 and 2 (Table 1, method: DB_ref_ + DB_novel_ + R_low_ + F_peptide+mRNA_), 77682 peptides were identified and this is 8.43% more peptide identification than the standard approach (71645 peptides) and 7.47% more peptide identification than step 1 on Jurkat cell line dataset. There were 53024 peptides identified and this is 6.18% more peptide identification than the standard approach (49937 peptides) and 3.98% more peptide identification than step 1 on mouse liver dataset. The results indicated that step 2 is more important than step 1.

### Permutation test for the features taken for improvement of peptide identification

**Fig. 5:**
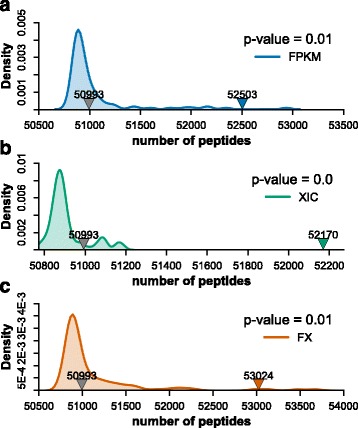
Validation by permutation test. The *left* and *right arrows* indicate the peptides numbers of MascotPercolator (*left arrow*) and (**a**) adding FPKM (*right arrow*), (**b**) XIC (*right arrow*), and (**c**) both (*right arrow*) as features. The *p*-value was calculated by permutation test (100 times)

In order to ensure the authenticity for the expanded identifications due to addition of the features, we conducted a permutation test (100 times) by shuffling FPKM and XIC assignment for peptide identification in Percolator processing. In this permutation test, the *p*-value was calculated as “(the number of peptide identification greater than that from the non-shuffling features processing)/(the times for permutation)”. The test results shown in Fig. 5 revealed that the *p*-values for addition of FPKM, XIC, or both features were all less than 0.05, indicating the enhancement of peptide identification was truly dependent upon the specific feature but not the order of adding feature.

## Conclusions

Using RNA-Seq data including its qualitative and quantitative information is reasoned a promising strategy to improve the sensitivity of peptide identifications and identify novel peptides in proteomic analysis on the basis of MS/MS data. In this study, we described an approach how to integrate the post-processing algorithm with the RNA-Seq information for improving the sensitivity and accuracy of peptide identification. With incorporating of the transcript and peptide abundance as the feature to rescore PSMs during peptide searching, we demonstrated that this approach could significantly improve the sensitivity in peptide identification and novel peptide detection.

## Additional files

Additional file 1: Scripts used for data analysis in this study. (DOCX 35 kb)
Additional file 2: Supplementary Method. The calculation of the XIC of peptides. (DOCX 21 kb)
Additional file 3: Figure S1. The weights of features for (A) Jurcat cell line and (B) mouse liver datasets. (DOCX 140 kb)
Additional file 4: Table S1. Overview of the two datasets in RNA-Seq level. (DOCX 19 kb)
Additional file 5: Figure S2. The quality check of uniquely identified 402 peptides by the method with adding MS1 XIC feature but not for the method with adding both FPKM and MS1 XIC features and a comparison of Mascot scores for peptide identification towards all the peptides identified in the dataset was presented. (DOCX 82 kb)
Additional file 6: Table S2. Summary of spectrum identification with 1% FDR in PSM level for different methods on two data sets. (DOCX 15 kb)

## Abbreviations

FDR: False discovery rate
FPKM: Fragments Per Kilobase of transcript per Million mapped reads
MS: Mass spectrometry
PSM: Peptide spectrum match
XIC: Extracted ion current
