# Supplementary material for: Improvement of peptide identification with considering the abundance of mRNA and peptide
Source: BMC Bioinformatics. 2017 Feb 16;18:109. doi: 10.1186/s12859-017-1491-5 (PMC5311845; doi:10.1186/s12859-017-1491-5)
Supplement: Additional file 1: — Scripts used for data analysis in this study. (DOCX 35 kb) [file 12859_2017_1491_MOESM1_ESM.docx]

**Analysis Scripts**

1. **RNA-Seq analysis for Jurkat cell line data**

| **run_qc_1.sh** |
| --- |
| filter_fq_V6 \  -u 33 \  --fq1 SRR791578_1.fq.gz \  --fq2 SRR791578_2.fq.gz \  -N 0.1 \  -A 1 \  -Q 5,0.5 \  --qual 33 \  --Gnum 100 \  --out clean_78 \  --type 1 \  --plot gnuplot |
| **run_QC_2.sh** |
| filter_fq_V6 \  -u 33 \  --fq1 SRR791579_1.fq.gz \  --fq2 SRR791579_2.fq.gz \  -N 0.1 \  -A 1 \  -Q 5,0.5 \  --qual 33 \  --Gnum 100 \  --out clean_79 \  --type 1 \  --plot gnuplot |
| **run_QC_3.sh** |
| filter_fq_V6 \  -u 33 \  --fq1 SRR791580_1.fq.gz \  --fq2 SRR791580_2.fq.gz \  -N 0.1 \  -A 1 \  -Q 5,0.5 \  --qual 33 \  --Gnum 100 \  --out clean_80 \  --type 1 \  --plot gnuplot |
| **tophat.sh** |
| tophat2 \  -p 6 \  --b2-very-sensitive \  --segment-mismatches 2 \  -r 150 \  --library-type fr-unstranded \  -G Homo_sapiens.GRCh37.75.exon.gtf \  -o tophat_clean \  Homo_sapiens.GRCh37.75.dna.toplevel \  clean_78_1.fq.gz,\  clean_79_1.fq.gz,\  clean_80_1.fq.gz \  clean_78_2.fq.gz,\  clean_79_2.fq.gz,\  clean_80_2.fq.gz |
| **cufflinks.sh** |
| cufflinks \  -u \  -p 6 \  -g Homo_sapiens.GRCh37.75.exon.gtf \  -b Homo_sapiens.GRCh37.75.dna.toplevel.fa \  -o cuff_g \  accepted_hits.sorted.bam |
| **cuffcompare.sh** |
| cuffcompare \  -V \  -o cmp \  -r Homo_sapiens.GRCh37.75.exon.gtf \  transcripts.gtf |

1. **RNA-Seq analysis for mouse liver data**

| **run_clean.sh** |
| --- |
| filter_fq_V6 \  -u 33 \  --fq1 Sam_011033694_L1_1.fq.gz \  --fq2 Sam_011033694_L1_2.fq.gz \  -N 0.1 \  -A 1 \  -Q 5,0.5 \  --qual 33 \  --Gnum 100 \  --out clean \  --type 1 \  --plot gnuplot |
| **tophat.sh** |
| tophat \  -p 8 \  -r 20 \  --solexa1.3-quals \  -G Mus_musculus.GRCm38.75.gtf \  -o tophat \  Mus_musculus.GRCm38.75.dna.toplevel \  clean_1.fq.gz \  clean_2.fq.gz |
| **cufflinks.sh** |
| cufflinks \  --max-bundle-length 10000000 \  -u \  -p 8 \  -g Mus_musculus.GRCm38.75.gtf \  -b Mus_musculus.GRCm38.75.dna.toplevel.fa \  -o ./ \  accepted_hits.bam |
| **cuffcompare.sh** |
| cuffcompare \  -V \  -r ref.gtf \  transcripts.gtf |

1. **Proteomics analysis for Jurkat cell line data**

| **asc_batch_creator.v1.0.sh** |
| --- |
| pro_database="JURKAT_1"  ascdir='./asc/'  if [ ! -d $ascdir ]; then  mkdir -p $ascdir  fi  mgfdir='./raw_data/'  function ergodic(){  for file in ` ls $1 \|perl -ne 'print if /.+.mgf$/' `  do  if [ -d $1$file ]  then  ergodic $1$file  else  local path_name=$1$file  local name=$file  echo $path_name  perl mascot_v1_2_0.pl \  -file $path_name \  -db $pro_database \  -tol 10 \  -tolu ppm \  -itol 0.05 \  -itolu Da \  -mods='Carbamidomethyl (C)' \  -mass=Monoisotopic \  -cle Trypsin \  -decoy=1 \  -charge='2+ and 3+' \  -pfa 2 \  -it_mods='Oxidation (M)' \  -instrument=Default \  -com Jurkat \  -outdir $ascdir  fi  done  }  ergodic $mgfdir |
| **qsub-sge4mascot_creator.sh** |
| #!/bin/bash -  ascdir='./asc'  export ascdir  datdir='./dat'  export datdir  if [ ! -d $datdir ]; then  mkdir -p $datdir  fi  ls $ascdir\|perl -MFile::Spec -ne 'chomp;print "cd ./Mascot_v2.3/cgi/;./nph-mascot.exe 1 -commandline -f ",File::Spec->rel2abs($ENV{"datdir"}),"/$_.dat < ",File::Spec->rel2abs($ENV{"ascdir"}),"/$_\n" if /asc$/'>run_mascot4sge2.sh |
| **run_mascot.sh** |
| for ((i=1;i<=28;i++))  do  sed -n ${i}'p' run_mascot4sge2.sh > "z"${i}".sh"  qsub –q q -P P -cwd -l vf=2G "z"${i}".sh"  done |
| **mascot_perculator.sh** |
| dat_dir='../dat/'  file_name=(`ls ${dat_dir} \| grep 'dat$'`)  out_dir='./out'  for ((i=0;i<${#file_name[@]};i++))  do  name=${dat_dir}"${file_name[$i]}"  out_name=${out_dir}${i}  echo $name  echo "java -cp MascotPercolator207.jar cli.MascotPercolator -decoy "${name}" -target "${name}" -features -out "${out_name} > "b"${i}".sh"  qsub -q q -P P -cwd -l vf=2G "b"${i}".sh"  done |
| **run_former.sh** |
| workdir="./"  #merge the fractions  echo "merge the 28 features fractions"  #python ${workdir}"merge_fea_file.py"  python_out=`python - <<EOF  #file: merge_fea_file.py  #!usr/bin/python  s1='./out'  s2='.features.txt'  #read the features in 28 different fractions  file_name=[]  for i in range(0,28):  file_name.append(s1+str(i)+s2)    #write features in the file  out=open('./features.txt',"w")  out.write("features:\n")  i=0  for files in file_name:  f=open(files,"r")  print files  line=f.readline()#the table head  while True:  line=f.readline()  if not line:  break  out.write(str(i)+line)  f.close()  i+=1    out.close()  EOF`  echo $python_out  #then have features.pep2  #the second step  echo "conduct the percolator program"  percolator -W config.default.feature -j ${workdir}"features.txt" -B ${workdir}"decoy.txt" -r ${workdir}"out.txt"  echo "done"  #end |

1. **Proteomics analysis for mouse liver data**

| **asc_batch_creator.v1.0.sh** |
| --- |
| pro_database="MOU1"  ascdir='./asc/'  if [ ! -d $ascdir ]; then  mkdir -p $ascdir  fi  mgfdir='./mgf/'  function ergodic(){  for file in ` ls $1 \|perl -ne 'print if /.+.mgf$/' `  do  if [ -d $1$file ]  then  ergodic $1$file  else  local path_name=$1$file  local name=$file  echo $path_name  perl mascot_v1_2_0.pl \  -file $path_name \  -db $pro_database \  -tol 10 \  -tolu ppm \  -itol 0.5 \  -itolu Da \  -mods='Carbamidomethyl (C)' \  -mass=Monoisotopic \  -cle Trypsin \  -decoy=1 \  -charge='2+, 3+ and 4+' \  -pfa 2 \  -it_mods='Oxidation (M)' \  -instrument=Default \  -com BPRC \  -outdir $ascdir  fi  done  }  ergodic $mgfdir |
| **qsub-sge4mascot_creator.sh** |
| #!/bin/bash -  ascdir='./asc'  export ascdir  datdir='./dat'  export datdir  if [ ! -d $datdir ]; then  mkdir -p $datdir  fi  ls $ascdir\|perl -MFile::Spec -ne 'chomp;print "cd ./Mascot_v2.3/cgi/;./nph-mascot.exe 1 -commandline -f ",File::Spec->rel2abs($ENV{"datdir"}),"/$_.dat < ",File::Spec->rel2abs($ENV{"ascdir"}),"/$_\n" if /asc$/'>run_mascot4sge2.sh |
| **run_mascot.sh** |
| for ((i=1;i<=28;i++))  do  sed -n ${i}'p' run_mascot4sge2.sh > "z"${i}".sh"  qsub -q q -P P -cwd -l vf=2G "z"${i}".sh"  done |
| **mascot_perculator.sh** |
| #!/bin/bash  dat_dir='../dat/'  file_name=(`ls ${dat_dir} \| grep 'dat$'`)  out_dir='./out'  for ((i=0;i<${#file_name[@]};i++))  do  name=${dat_dir}"${file_name[$i]}"  out_name=${out_dir}${i}  echo $name  echo "java -cp MascotPercolator207.jar cli.MascotPercolator -decoy "${name}" -target "${name}" -features -out "${out_name} > "b"${i}".sh"  qsub -q q -P P -cwd -l vf=2G "b"${i}".sh"  done |
| **run_former.sh** |
| workdir="./"  #merge the fractions  echo "merge the 52 features fractions"  #python ${workdir}"merge_fea_file.py"  python_out=`python - <<EOF  #file: merge_fea_file.py  #!usr/bin/python  s1='./out'  s2='.features.txt'  #read the features in 52 different fractions  file_name=[]  for i in range(0,52):  file_name.append(s1+str(i)+s2)    #write features in the file  out=open('./features.txt',"w")  out.write("features:\n")  i=0  for files in file_name:  f=open(files,"r")  print files  line=f.readline()#the table head  while True:  line=f.readline()  if not line:  break  out.write(str(i)+line)  f.close()  i+=1    out.close()  EOF`  echo $python_out  #the second step  echo "conduct the percolator program"  percolator -W config.default.feature -j ${workdir}"features.txt" -B ${workdir}"decoy.txt" -r ${workdir}"out.txt"  echo "done"  #end |

1. **Integrative analysis for jurkat cell line data**

| **yield_xic.sh** |
| --- |
| #!/bin/bash  dat_dir='./dat/'  dat_file_name=(`ls ${dat_dir} \| grep 'dat$'`)  mz_dir='./mzXML/'  mz_file_name=(`ls ${mz_dir}`)  out_dir='./xic_out'  for ((i=0;i<${#dat_file_name[@]};i++))  do  dat_name=${dat_dir}"${dat_file_name[$i]}"  zm_name=${mz_dir}"${mz_file_name[$i]}"  out_name=${out_dir}${i}  echo $dat_name  echo $zm_name  echo "java -jar xictool.jar -dat "${dat_name}" -ms "${zm_name}" -offset 90 -out "${out_dir}" -snr 1" > "xic"${i}".sh"  qsub -q q -P P -cwd -l vf=2G "xic"${i}".sh"  done |
| **add_xic.py** |
| import math  import sys  FILE_features_former=sys.argv[1]  FILE_pep_abundance=sys.argv[2]  FILE_features_new=sys.argv[3]  #construct the xic dictionary  xic_dict={}  file_pep=open(FILE_pep_abundance,"r")  file_pep.readline()  while True:  line=file_pep.readline()  if not line:  break  pro_index=line.split()[0]+line.split()[1] #id+label  f1=float(line.split()[9]) #xic1  f2=float(line.split()[10]) #xic2  xic_dict.setdefault(pro_index,[f1,f2]) #id+label(0),xic1,xic2  file_pep.close()  #construct end  print 'construct the xic dictionary end'  #add feature  file_feature_former=open(FILE_features_former,"r")  file_feature_new=open(FILE_features_new,"w")  #the first line  line=file_feature_former.readline()  #here, remained to be refined!  file_feature_new.write(line)  #protein_have_no_tran=0  #protein_have_tran=0  while True:  line=file_feature_former.readline()  if not line:  break  index_list=line.split()[0]+line.split()[1]#to find the protein id in the feature  file_feature_new.write(line.split('\t',2)[0])  file_feature_new.write('\t')  file_feature_new.write(line.split('\t',2)[1])  file_feature_new.write('\t')  if xic_dict.has_key(index_list):  #file_feature_new.write(str(math.log(1+tran_FPKM_dict[index_list][0])))  #file_feature_new.write('\t')#here, notice  file_feature_new.write(str(math.log(1+xic_dict[index_list][1]))) #xic2  file_feature_new.write('\t')#here, notice  else:  file_feature_new.write("0.0\t")  file_feature_new.write(line.split('\t',2)[2])  file_feature_former.close()  file_feature_new.close() |
| **add_xic.pepline.sh** |
| workdir="./"  featuredir="./mp/"  #the same order as dat dir and mzxml dir (0-41)  xicdir="./xic_out/"  xic_file_name=(`ls $xicdir \| grep 'txt$'`)  #add peptide abundance feature 2  #28 fraction totally  echo "add peptide abundance xic 2 to former 28 fractions"  for i in {0..27}  do  python_out=`python ./add_xic.py ${featuredir}"out"${i}".features.txt" ${xicdir}"${xic_file_name[$i]}" ${workdir}"frac"${i}".features.xic.txt"`  echo $python_out  done  #merge the fractions  echo "merge the 28 features fractions"  #python ${workdir}"merge_fea_file.py"  python_out=`python - <<EOF  #file: merge_fea_file.py  #!usr/bin/python  s1='./frac'  s2='.features.xic.txt'  #read the features in 28 different fractions  file_name=[]  for i in range(0,28):  file_name.append(s1+str(i)+s2)    #write features in the file  out=open('./features.xic',"w")  out.write("features:\n")  i=0  for files in file_name:  f=open(files,"r")  print files  line=f.readline()#the table head  while True:  line=f.readline()  if not line:  break  out.write(str(i)+line)  f.close()  i+=1    out.close()  EOF`  echo $python_out  #then have features.pep2  #the second step  echo "conduct the percolator program"  percolator -W config.default.feature -j ${workdir}"features.xic" -B decoy.txt -r ${workdir}"out.xic"  echo "done"  #end |
| **add_fpkm.awk** |
| NR==FNR{if(NR!=1)fpkm[$1]=$2}  NR!=FNR{  i=51;max=0;line=$0;  while(i<=NF){  split($i,b,"_");  id=b[1];  if(fpkm[id]>max){  max=fpkm[id]  }  ++i  }  j=1;  while(j<=2){  printf("%s\t",$j)  ++j  }  printf("%s\t",log(max+1))  while(j<=NF){  printf("%s\t",$j)  ++j  }  printf("\n")  } |
| **fpkm.sh** |
| percolator -W config.default.feature -j feature.fpkm -B tab.txt -r out.fpkm |
| **add_xic_fpkm.awk** |
| NR==FNR{if(NR!=1)fpkm[$1]=$2}  NR!=FNR{  i=52;max=0;line=$0;  while(i<=NF){  split($i,b,"_");  id=b[1];  if(fpkm[id]>max){  max=fpkm[id]  }  ++i  }  j=1;  while(j<=2){  printf("%s\t",$j)  ++j  }  printf("%s\t",log(max+1))  while(j<=NF){  printf("%s\t",$j)  ++j  }  printf("\n")  } |
| **fpkm_xic.sh** |
| percolator -W config.default.feature -j feature.xic.fpkm.0.1 -B tab.txt -r out.xic.fpkm |

1. **Integrative analysis for mouse liver data**

| **yield_xic.sh** |
| --- |
| #!/bin/bash  dat_dir='./dat/'  dat_file_name=(`ls ${dat_dir} \| grep 'dat$'`)  mz_dir='./mzXML/'  mz_file_name=(`ls ${mz_dir}`)  out_dir='./xic_out'  for ((i=0;i<${#dat_file_name[@]};i++))  do  dat_name=${dat_dir}"${dat_file_name[$i]}"  zm_name=${mz_dir}"${mz_file_name[$i]}"  out_name=${out_dir}${i}  echo $dat_name  echo $zm_name  echo "java -Xms4096m -Xmx4096m -jar xictool.jar -dat "${dat_name}" -ms "${zm_name}" -offset 90 -out "${out_dir}" -snr 1" > "xic"${i}".sh"  qsub -q q -P P -cwd -l vf=4G "xic"${i}".sh"  done |
| **add_xic.py** |
| import math  import sys  FILE_features_former=sys.argv[1]  FILE_pep_abundance=sys.argv[2]  FILE_features_new=sys.argv[3]  #construct the xic dictionary  xic_dict={}  file_pep=open(FILE_pep_abundance,"r")  file_pep.readline()  while True:  line=file_pep.readline()  if not line:  break  pro_index=line.split()[0]+line.split()[1] #id+label  f1=float(line.split()[9]) #xic1  f2=float(line.split()[10]) #xic2  xic_dict.setdefault(pro_index,[f1,f2]) #id+label(0),xic1,xic2  file_pep.close()  #construct end  print 'construct the xic dictionary end'  #add feature  file_feature_former=open(FILE_features_former,"r")  file_feature_new=open(FILE_features_new,"w")  #the first line  line=file_feature_former.readline()  #here, remained to be refined!  file_feature_new.write(line)  #protein_have_no_tran=0  #protein_have_tran=0  while True:  line=file_feature_former.readline()  if not line:  break  index_list=line.split()[0]+line.split()[1]#to find the protein id in the feature  file_feature_new.write(line.split('\t',2)[0])  file_feature_new.write('\t')  file_feature_new.write(line.split('\t',2)[1])  file_feature_new.write('\t')  if xic_dict.has_key(index_list):  #file_feature_new.write(str(math.log(1+tran_FPKM_dict[index_list][0])))  #file_feature_new.write('\t')#here, notice  file_feature_new.write(str(math.log(1+xic_dict[index_list][1]))) #xic2  file_feature_new.write('\t')#here, notice  else:  file_feature_new.write("0.0\t")  file_feature_new.write(line.split('\t',2)[2])  file_feature_former.close()  file_feature_new.close() |
| **add_xic.pepline.sh** |
| workdir="./"  featuredir="./mp/"  #the same order as dat dir and mzxml dir (0-41)  xicdir="./xic_out/"  xic_file_name=(`ls $xicdir \| grep 'txt$'`)  #add peptide abundance feature 2  #52 fraction totally  echo "add peptide abundance xic 2 to former 52 fractions"  for i in {0..51}  do  python_out=`python ./add_xic.py ${featuredir}"out"${i}".features.txt" ${xicdir}"${xic_file_name[$i]}" ${workdir}"frac"${i}".features.xic.txt"`  echo $python_out  done  #merge the fractions  echo "merge the 52 features fractions"  #python ${workdir}"merge_fea_file.py"  python_out=`python - <<EOF  #file: merge_fea_file.py  #!usr/bin/python  s1='./frac'  s2='.features.xic.txt'  #read the features in 52 different fractions  file_name=[]  for i in range(0,52):  file_name.append(s1+str(i)+s2)    #write features in the file  out=open('./features.xic',"w")  out.write("features:\n")  i=0  for files in file_name:  f=open(files,"r")  print files  line=f.readline()#the table head  while True:  line=f.readline()  if not line:  break  out.write(str(i)+line)  f.close()  i+=1    out.close()  EOF`  echo $python_out  #then have features.pep2    #the second step  echo "conduct the percolator program"  percolator -W config.default.feature -j ${workdir}"features.xic" -B decoy.xic -r ${workdir}"out.xic"  echo "done"  #end |
| **add_fpkm.awk** |
| NR==FNR{if(NR!=1)fpkm[$1]=$2}  NR!=FNR{  i=51;max=0;line=$0;  while(i<=NF){  split($i,b,"_");  id=b[1];  if(fpkm[id]>max){  max=fpkm[id]  }  ++i  }  j=1;  while(j<=2){  printf("%s\t",$j)  ++j  }  printf("%s\t",log(max+1))  while(j<=NF){  printf("%s\t",$j)  ++j  }  printf("\n")  } |
| **fpkm.sh** |
| percolator -W config.default.feature -j feature.fpkm -B decoy.fpkm -r out.fpkm |
| **add_xic_fpkm.awk** |
| NR==FNR{if(NR!=1)fpkm[$1]=$2}  NR!=FNR{  i=52;max=0;line=$0;  while(i<=NF){  split($i,b,"_");  id=b[1];  if(fpkm[id]>max){  max=fpkm[id]  }  ++i  }  j=1;  while(j<=2){  printf("%s\t",$j)  ++j  }  printf("%s\t",log(max+1))  while(j<=NF){  printf("%s\t",$j)  ++j  }  printf("\n")  } |
| **fpkm.xic.sh** |
| percolator -W config.default.feature -j f0.1.xic.fpkm.features -B decoy.xic.fpkm -r out.xic.fpkm |
