# Supplementary material for: Improvement of peptide identification with considering the abundance of mRNA and peptide
Source: BMC Bioinformatics. 2017 Feb 16;18:109. doi: 10.1186/s12859-017-1491-5 (PMC5311845; doi:10.1186/s12859-017-1491-5)
Supplement: Additional file 2: — Supplementary Method. The calculation of the XIC of peptides. (DOCX 21 kb) [file 12859_2017_1491_MOESM2_ESM.docx]

**Supplementary Method**

**The calculation of the XIC of peptides**

In order for peptide label-free quantification, we developed a java-based cross-platform software named XQuant that can conduct XIC extraction, retention time (RT) alignment for multiple runs, protein inference, and protein quantification, etc. Herein we provide a brief description of how XQuant calculates XIC as peptide abundance.

Mass spectrometry experiments of complex biological samples are typically divided into tens of fractions (28 fractions for jurkat cell line and 52 fractions for mouse liver), for each of which, XQuant firstly extracts out the MS1 information from MzXML/MzML format file into temporary directory. In this processing, XQuant will estimate the peak type of the spectrum based on the variance of inter-peak distances to determine ‘centroid’ or ‘profile’ spectrum type, if the latter is specified, the spectra will be converted to centroid type before being stored.

Then, in a specified fraction, all of the peptides, exactly PSMs, are quantified. All peptides are sorted with regard to their retention times. For a target peptide to be quantify, all the corresponding MS1 spectrum within a specific RT range will be extracted by a window sliding along all the MS1 spectrum of such fraction. Note that user-specific minimum and maximum RT and m/z will be used to reduce the iteration time.

Afterwards, for a specific peptide, its theoretical isotope pattern was calculated. If the number of calculated peaks exceeds three, then the ones with relative intensities less than 0.1 are to be cut off, but only up to four isotopic peaks are allowed. Furthermore, the peptide modification and neutral loss are also concerned in isotope pattern calculation.

The XIC values are calculated by m/z trace and peak detection algorithm:

1. For a specific isotope peak, all the ion peaks within m/z tolerance and RT tolerance was extracted, forming a mass trace.
2. Within such mass trace, all possible elution peaks are detected. The elution peak, whose centroid retention time is the closest to the target peptide’s retention time, is the peptide’s XIC that we want.
3. After extracting the elution peak for each isotope peak, the area under the curve is calculated as abundance quantity.
4. Among the isotope pattern, the mono-isotopic mass is the most abundant isotopic mass. And the XIC corresponding to mono-isotopic mass is used as our final peptide quantification value.
