# Supplementary figures and images for: Improvement of peptide identification with considering the abundance of mRNA and peptide
Source: BMC Bioinformatics. 2017 Feb 16;18:109. doi: 10.1186/s12859-017-1491-5 (PMC5311845; doi:10.1186/s12859-017-1491-5)

**
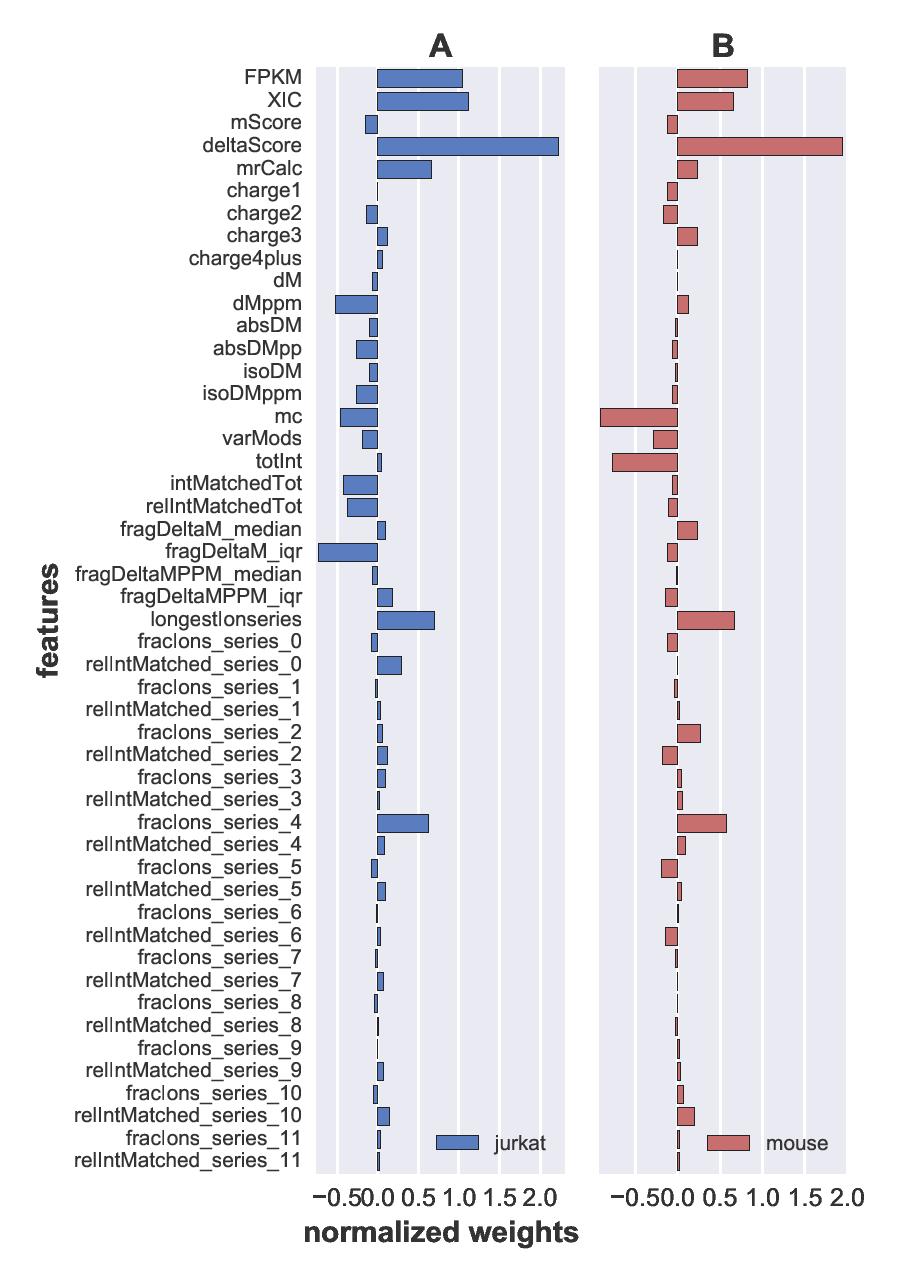
**

**Figure S1**. The weights of features for (A) Jurcat cell line and (B) mouse liver datasets.

Supplement: Additional file 3: Figure S1. — The weights of features for (A) Jurcat cell line and (B) mouse liver datasets. (DOCX 140 kb) [file 12859_2017_1491_MOESM3_ESM.docx]
