# Supplementary material for: Improvement of peptide identification with considering the abundance of mRNA and peptide
Source: BMC Bioinformatics. 2017 Feb 16;18:109. doi: 10.1186/s12859-017-1491-5 (PMC5311845; doi:10.1186/s12859-017-1491-5)
Supplement: Additional file 4: Table S1. — Overview of the two datasets in RNA-Seq level. (DOCX 19 kb) [file 12859_2017_1491_MOESM4_ESM.docx]

**Table S1.** Overview of two two datasets in RNA-Seq level.

|  | **Reads** | **Aligned reads** | **Alignment rate** |
| --- | --- | --- | --- |
| Jurkat cell line | 70921586 | 64116149 | 90.40% |
| Mouse liver | 37457172 | 33747349 | 90.10% |
