# Supplementary material for: Improvement of peptide identification with considering the abundance of mRNA and peptide
Source: BMC Bioinformatics. 2017 Feb 16;18:109. doi: 10.1186/s12859-017-1491-5 (PMC5311845; doi:10.1186/s12859-017-1491-5)
Supplement: Additional file 5: Figure S2. — The quality check of uniquely identified 402 peptides by the method with adding MS1 XIC feature but not for the method with adding both FPKM and MS1 XIC features and a comparison of Mascot scores for peptide identification towards all the peptides identified in the dataset was presented. (DOCX 82 kb) [file 12859_2017_1491_MOESM5_ESM.docx]

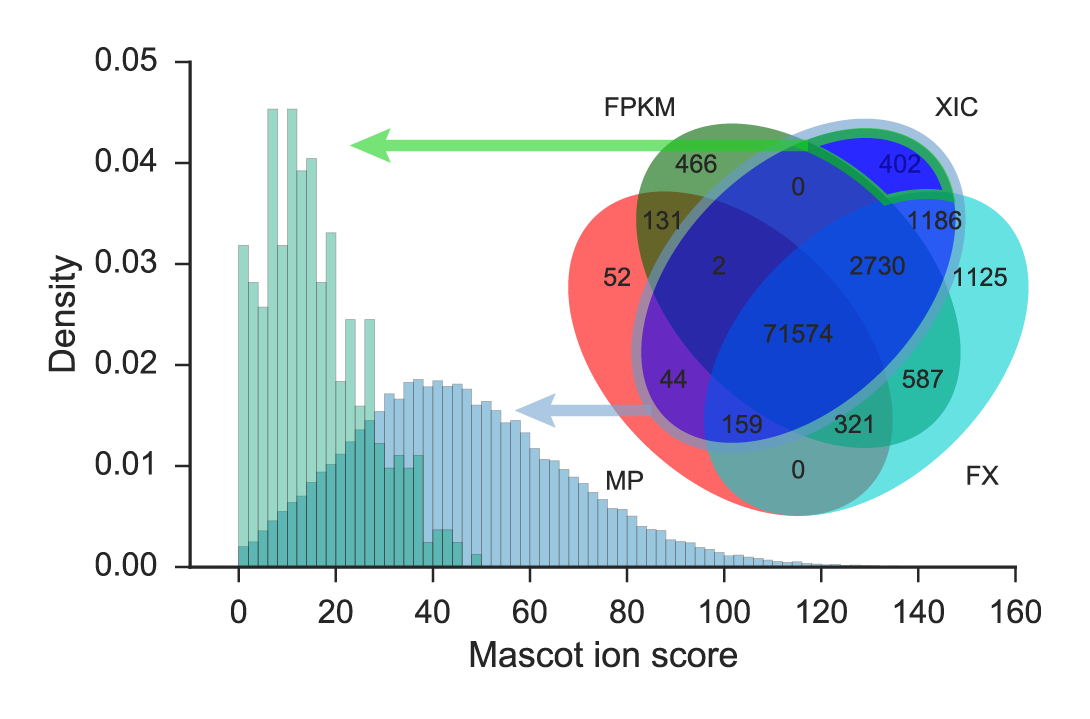


**Figure S2**. The quality check of uniquely identified 402 peptides by the method with adding MS1 XIC feature but not for the method with adding both FPKM and MS1 XIC features and a comparison of Mascot scores for peptide identification towards all the peptides identified in the dataset was presented
