# Supplementary material for: Improvement of peptide identification with considering the abundance of mRNA and peptide
Source: BMC Bioinformatics. 2017 Feb 16;18:109. doi: 10.1186/s12859-017-1491-5 (PMC5311845; doi:10.1186/s12859-017-1491-5)
Supplement: Additional file 6: Table S2. — Summary of spectrum identification with 1% FDR in PSM level for different methods on two data sets. (DOCX 15 kb) [file 12859_2017_1491_MOESM6_ESM.docx]

Table S2. Summary of spectrum identification with 1% FDR in PSM level for different methods on two data sets.

| **Methods** | **Jurkat cell line**  **(483971 spectra in total)** | | **Mouse liver**  **(3997752 spectra in total)** | |
| --- | --- | --- | --- | --- |
|  | **PSMs** | **Ratio^b^** | **PSMs** | **Ratio** |
| DB_ref_+DB_novel_ | 225346 (46.56%^a^) | - | 422475 (10.57%) | - |
| DB_ref_+DB_novel_+R_low_ | 225732 (46.64%) | 0.17% | 431956 (10.80%) | 2.24% |
| DB_ref_+DB_novel_+R_low_+F_mRNA_ | 237776 (49.13%) | 5.52% | 461560 (11.55%) | 9.25% |
| DB_ref_+DB_novel_+R_low_+F_peptide_ | 236892 (48.95%) | 5.12% | 441091 (11.03%) | 4.41% |
| DB_ref_+DB_novel_+R_low_+F_peptide+mRNA_ | 242493 (50.10%) | 7.61% | 466007 (11.66%) | 10.30% |

^a^. Spectra identification successful rate.

^b^. The improvement ratio compared with the method “DB_ref_+DB_novel_”
